# Supplementary material for: Childbirth Satisfaction during the COVID-19 Pandemic in a Hospital in Southwestern Spain
Source: Int J Environ Res Public Health. 2022 Aug 5;19(15):9636. doi: 10.3390/ijerph19159636 (PMC9367768; doi:10.3390/ijerph19159636)
Supplement: Supplementary file 1 [file ijerph-19-09636-s001.zip › ijerph-1816549-supplementary.pdf]

Supplementary File S1. Women excluded vs women included in the final analysis.

Variables	Women Included n= 116	Women excluded n=30	p
**Age x̄ (SD)**	32.08 (4.68)	32.09 (6.23)	0.993 [1]
**Country of origin n (%)**			
Spain	109 (94.0)	26 (86,7)	0.369 [2]
Other	7 (6.0)	4 (13.3)	
**Education n (%)**			
No studies/primary	40 (34.5)	10 (33.3)	0.234 [3]
Secondary	53 (45.7)	7 (23.3)	
University/postgraduate	23 (19.8)	13 (43.3)	
**Employment situation n (%)**			
Student	2 (1.7)	0 (0)	0.573 [2]
Unemployed	41 (35.3)	14 (46.7)	
Work for others	65 (56.0)	16 (53.3)	
Own-account work	8 (6.9)	0 (0)	
**Stable partner n (%)**	116 (100.0)	30 (100)	1.000 [3]
**Maternal Education n (%)**			
Yes	46 (39.7)	12 (40.0)	0.963 [3]
No	70 (60.3)	18 (60.0)	
**Number of professionals who provided care, n (%)**			
1 - 2	37 (31.9)	13 (43.3)	0.329 [2]
3 - 5	67 (57.8)	13 (43.3)	
> 5	26 (10.4)	4 (13.3)	
**Number of professionals who performed vaginal examination, n (%)**			
0 - 2	63 (54.3)	16 (53.3)	0.852 [2]
3 - 5	52 (44.8)	14 (46.7)	
> 5	1 (0.9)	0 (0)	
**Labor Companion, n (%)**			
Partner	103 (88.8)	23 (76.7)	0.092 [2]
Other relative	4 (3.4)	0 (0)	
Nobody	9 (7.8)	7 (23.3)	
**Puerperium Companion, n (%)**			
Partner	111 (95.7)	27 (90.0)	0.327 [2]
Other relative	2 (1.7)	0 (0)	
Nobody	3 (2.6)	3 (10.0)	
**The companion was chosen prior to childbirth, n (%)**	104 (89.7)	30 (100)	0.460 [3]
**Planned epidural analgesia before childbirth, n (%)**	85 (73.3)	24 (80.0)	0.618 [3]
**Planned mode of new-born feeding (prior to birth)**			
Exclusive breastfeeding	91 (78.4)	23 (76.7)	0.653 [2]
Partial breastfeeding	11 (9.5)	2 (6.7)	
Formula feeding	14 (12.1)	5 (16.6)	

[1] t-student test; [2] Fisher test; [3] Chi square test
